# Unravelling selection signatures in a single dog breed suggests recent selection for morphological and behavioral traits

**DOI:** 10.1002/ggn2.10024

**Published:** 2020-08-10

**Authors:** Juliane Friedrich, Andrea Talenti, Per Arvelius, Erling Strandberg, Marie J. Haskell, Pamela Wiener

**Affiliations:** ^1^ Division of Genetics and Genomics The Roslin Institute and Royal (Dick) School of Veterinary Studies, University of Edinburgh Midlothian UK; ^2^ Swedish Armed Forces Dog Training Center Märsta Sweden; ^3^ Department of Animal Breeding and Genetics Swedish University of Agricultural Sciences Uppsala Sweden; ^4^ Animal & Veterinary Sciences Scotland's Rural College (SRUC) Edinburgh UK

**Keywords:** association analysis, coat color, dog behavior, German Shepherd dog, positive selection

## Abstract

Strong selection has resulted in substantial morphological and behavioral diversity across modern dog breeds, which makes dogs interesting model animals to study the underlying genetic architecture of these traits. However, results from between‐breed analyses may confound selection signatures for behavior and morphological features that were coselected during breed development. In this study, we assess population genetic differences in a unique resource of dogs of the same breed but with systematic behavioral selection in only one population. We exploit these different breeding backgrounds to identify signatures of recent selection. Selection signatures within populations were found on chromosomes 4 and 19, with the strongest signals in behavior‐related genes. Regions showing strong signals of divergent selection were located on chromosomes 1, 24, and 32, and include candidate genes for both physical features and behavior. Some of the selection signatures appear to be driven by loci associated with coat color (Chr 24; *ASIP*) and length (Chr 32; *FGF5*), while others showed evidence of association with behavior. Our findings suggest that signatures of selection within dog breeds have been driven by selection for morphology and behavior. Furthermore, we demonstrate that combining selection scans with association analyses is effective for dissecting the traits under selection.

## Graphical abstract

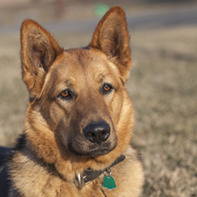

## 1 INTRODUCTION

The development of current dog breeds can be viewed as a unique long‐term selection experiment to study the process of domestication^1^ as well as short‐term evolutionary change as a consequence of intensive breeding.^2^ While the domestication of the modern dog (*Canis lupus familiaris*) from wolves took place at least 15 000 years ago,^3^ with some estimates considerably earlier (eg, 20 000 to 40 000 years ago^4^), the popularity of dogs has led to ongoing strict selection according to breeding schemes and standards imposed by breed associations and national kennel clubs. The establishment of genetically and phenotypically distinctive breeds by this intense artificial selection pressure has resulted in high intraspecies variation for physical and physiological features, disease susceptibility and behavior traits,^5^, ^6^, ^7^ which makes dogs powerful models to investigate the underlying genetic architecture and signatures of selection for various traits.

Genetic manifestation of the development of dog breeds can be seen as selection signatures, genomic regions targeted by natural or artificial selection that exhibit various characteristics, including population differentiation, extreme linkage disequilibrium (LD) and patterns of the haplotype structure (eg, long‐range haplotypes) or mutations in coding region.^8^ Accordingly, selection signatures between dog breeds have been reported for physical traits, domestication‐related traits and some specific behaviors and have led to the identification of candidate genes, for example, *IGF1* for body size, *FGF5* for coat length and *HAS2* for skin wrinkling,^2^ *AMY2B*, *MGAM* and *SGLT1* for adaptation to a starch‐rich diet^9^ and *TRPM3* and *ROBO1* for athletic success in sport‐hunting.^10^ In a recent whole‐genome sequence study of 144 modern dog breeds, positive human‐imposed selection was implicated in the fixation or high prevalence within breeds of a range of morphological characteristics (eg, ear shape, height, weight).^11^ These recent studies for selection signatures in dogs have focused on between‐breed or dog‐wolf comparisons and while such studies have allowed detection of signatures related to notable physical features, signatures for more subtle traits like behavior characteristics may be confounded with or masked by signals for the physical features, which might complicate the interpretation of these signatures as appears to be the case for association signals.^12^

In this study, we analyzed a single dog breed, the German Shepherd dog (GSD), to detect signals of selection. The breed was established in the late 19th century by crossing multiple breeds, with the initial purpose of creating a sheep‐herding dog^13^ and later use as a general working dog within the military or police. GSDs used in this study originated from two populations, the UK and Sweden; while the UK population represented a random sample of pet, show and working dogs, the Swedish dogs were bred within a breeding program of the Swedish Armed Forces (SAF) and only dogs that pass a behavior test can become working dogs or be used for breeding. Accordingly, in a previous study^14^ we showed that there were significant differences between the two GSD populations for various behavior traits as measured in a questionnaire, for example, aggression against strangers or dogs, chasing, and playfulness. In contrast, morphological differences between populations were reduced compared to between‐breed studies. We hypothesize that by comparing populations of the same breed but with different behavior‐related selection strategies, we may be able to identify selection signatures for behavior as well as those for physical traits. Furthermore, by applying multiple statistical tests for the detection of selection signatures, we have increased the power to detect true signals of selection. Nonetheless, despite the within‐breed approach, one of the main difficulties that remains is the identification of the actual trait(s) under selection. We addressed this issue by characterizing the relationship between selection signatures and statistical associations between genotype and phenotype (behavior and morphological traits) from the same populations. We suggest that this approach, combining population genetics and quantitative genetics methods, may also be applicable in other contexts.

## 2 RESULTS AND DISCUSSION

### 2.1 Genomic structure of populations

**FIGURE 1:**
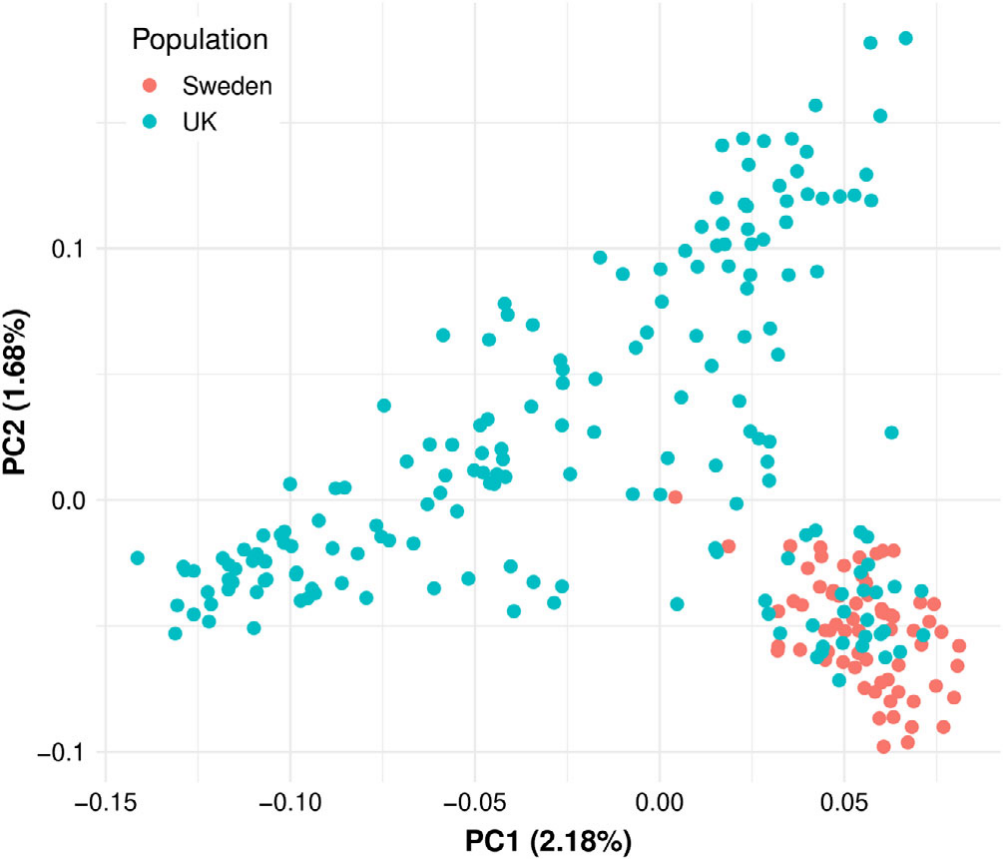
Principal Component Analysis of the pruned genomic data. Eigenvectors for the first two principal components are plotted and individuals are colored according to the population of origin. The variances explained by the principal components are given in parentheses

**FIGURE 2:**
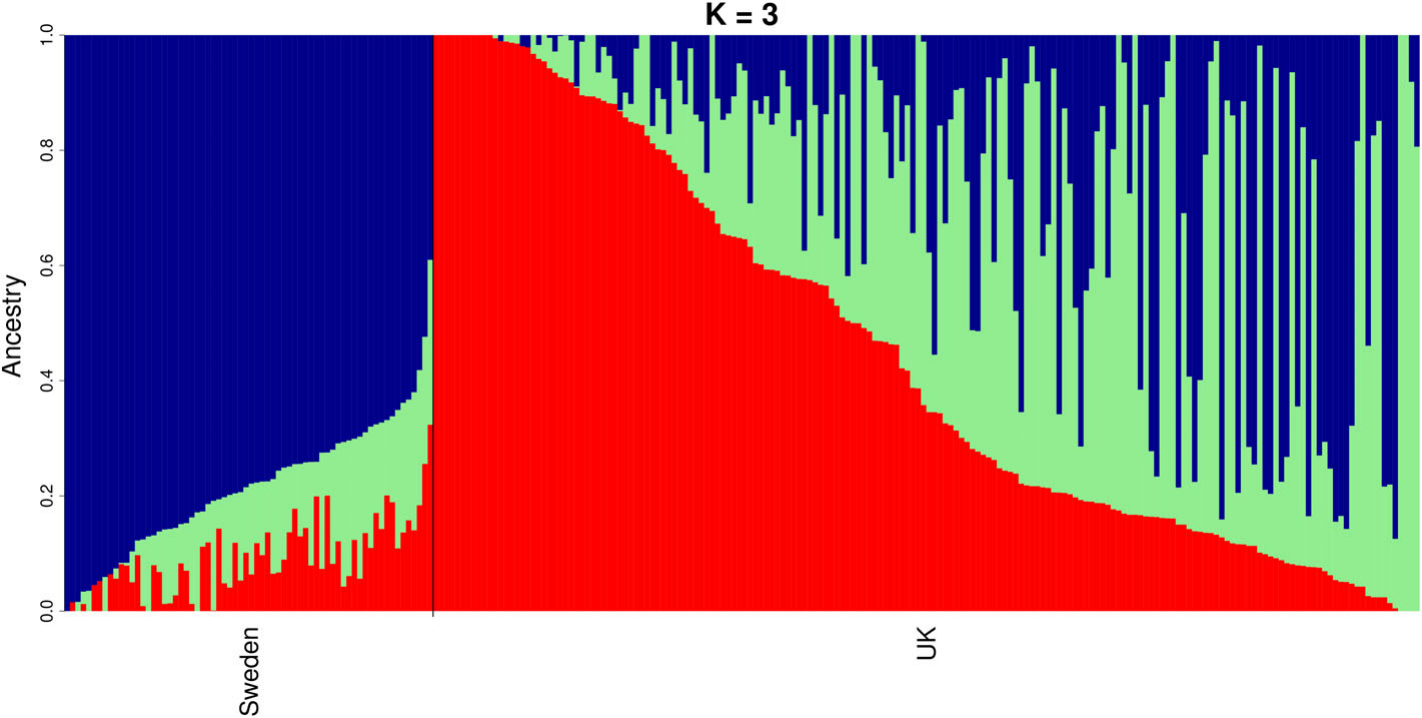
Ancestry proportions of studied GSDs based on the pruned genomic data assuming three underlying ancestries (K = 3 clusters) as revealed by ADMIXTURE. Each cluster is represented by a color and the length of the specific colored segment indicates the dog's proportion of membership in that cluster

Characterizing the genetic relationships between individual dogs is a valuable tool to evaluate the genetic structure of GSDs in this study. The underlying population structure in the two GSD populations (250 dogs in total) was explored by applying a principal component analysis (PCA) and ancestry estimation on a pruned SNP data set. The PCA indicated a separation between the UK and Swedish populations based on the first two principal components (PCs), which explained 2.8% and 1.9% of the genetic variance, respectively (Figure 1). With respect to PC1 and PC2, the UK dogs had a broader distribution than the Swedish GSDs, suggesting a stronger founder effect in the Swedish cohort. However, some of the UK GSDs clustered with the Swedish GSDs. The overall separation of the two populations is likely due to the geographical separation and thus primarily independent pedigrees but may also reflect the more recent origins of the Swedish population, with the SAF as the only breeder and the primary goal to breed good working dogs. The partial overlap between the two populations is likely due to the use of external dogs in the SAF breeding program, leading to some shared ancestry. A visual assessment of the ancestry estimation based on the ADMIXTURE program^15^ (Figure 2) also revealed a clear discrimination between the UK and Swedish populations. The lowest cross‐validation error of 0.55 was identified for K = 3 clusters (K = 3), with the blue cluster primarily associated with the Swedish population and the red and green clusters primarily associated with the UK population.

The average inbreeding coefficient calculated based on runs of homozygosity (F_ROH_) was 0.29 ± 0.02 (SD; SD) for Swedish GSDs and 0.31 ± 0.05 for UK GSDs. The significantly lower inbreeding estimate (*P* < .05) in the Swedish population might be a consequence of a strategic breeding scheme by the SAF. The average nucleotide diversity (μ) was 0.30 ± 0.16 for both populations.

### 2.2 Selection signatures within populations

**FIGURE 3:**
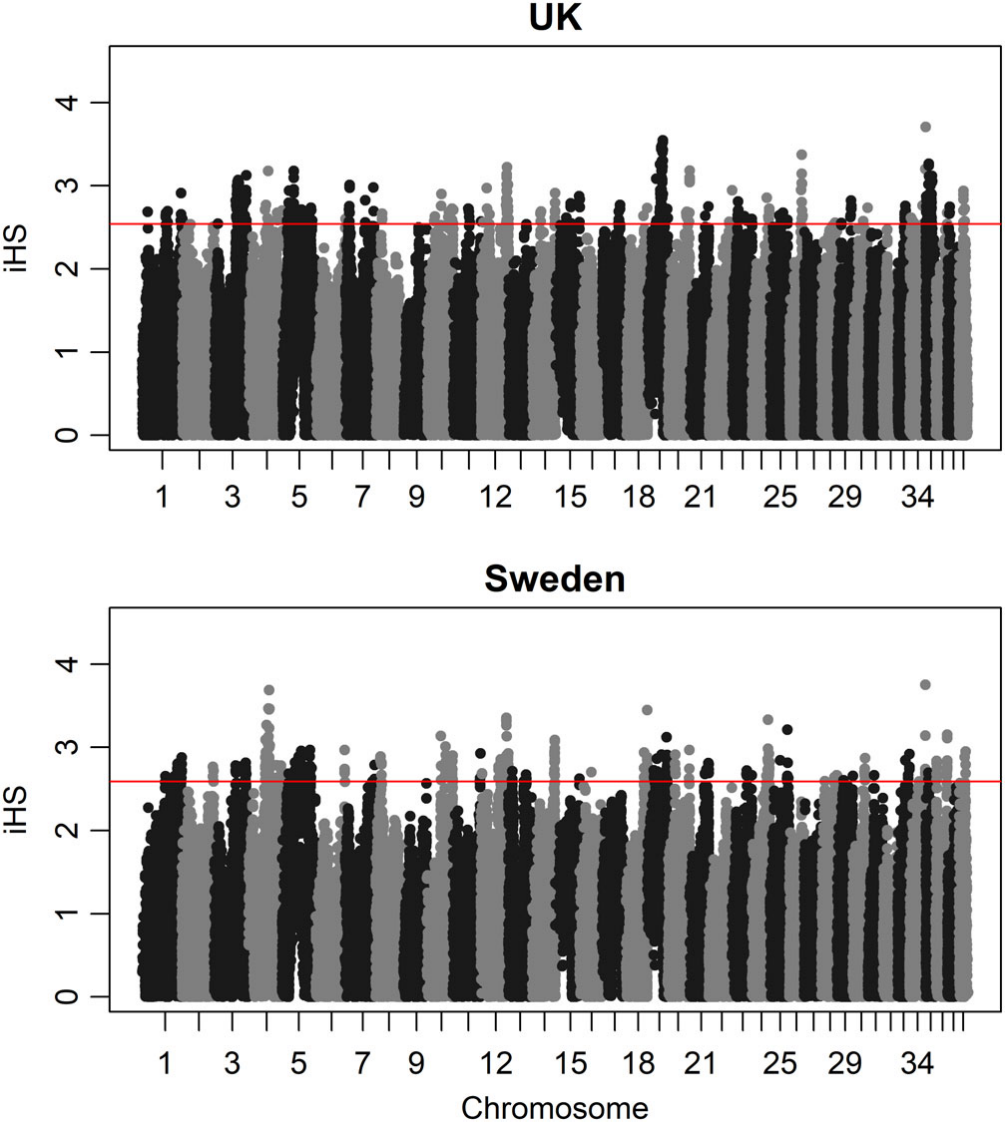
Distribution of integrated haplotype score (iHS) in the UK (upper plot) and Swedish population (lower plot). The red line indicates the threshold for the top 0.5% iHS

Selection signatures can be detected within populations by identifying distinctive patterns of LD. In the event of selective sweeps, favorable genetic variants increase in frequency and form extended haplotypes with neighboring genomic regions due to LD, as reviewed in Reference 16. We computed the integrated haplotype score (iHS), which is a variation of the extended haplotype homozygosity (EHH) statistic that aims to detect recent and incomplete selective sweeps within populations.^17^ In total, 197 and 142 regions with extreme EHH were detected within the UK and Swedish GSD population, respectively. A list of SNPs belonging to the top 0.5% of the iHS statistic in the UK and Swedish populations is given in Table S2. The iHS statistic identified similar selection signatures in both populations, but the most extreme values differed between populations, as shown by the 10 regions with the highest iHS statistics (Figure 3, Table 1). Regions with the highest iHS for the UK population were located on Chr 19 at 36.0 to 36.5 Mb and 37.5 to 37.7 Mb. A single marker on Chr 4 at 52.5 Mb showed the highest iHS in the Swedish population, followed by a region on Chr 18 at 54.9 to 55.3 Mb. The SNPs identified by iHS were further tested for their association with different traits (coat color, coat length, and behavior) separately for each population to identify the putative trait under selection.

**TABLE 1:** Top selection signatures within the UK and Swedish GSD populations, showing the 10 highest integrated haplotype score (iHS) statistics

Chr	Start (Mb)	Stop (Mb)	Distance (Mb)	N_SNPs_ ^a^	iHS peak^b^	iHS mean^c^	Gene(s)^d^	Phenotypic association^e^	
*UK population*								
5	29.2	29.8	0.62	16	3.18	2.84	*ENSCAFG00000015899*; *MMP20*; *MMP27*; *MMP7*; ***ENSCAFG00000030873***; ***BIRC2***; *BIRC3*; ***YAP1***; ***C11orf70***; *CEP126*; *ANGPTL5*	‐	
12	68.1	68.2	0.06	2	3.22	2.96	***TRAF3IP2***	‐	
19	33.0	33.1	0.04	4	3.26	2.84	n.a.	‐	
19	36.0	36.5	0.51	10	3.46	2.93	***NCKAP5***	‐	
19	36.8	37.0	0.19	5	3.18	2.90	n.a.	‐	
19	37.5	37.7	0.20	6	3.48	3.19	***TMEM163***	‐	
19	38.3	38.6	0.31	9	3.19	2.79	***ZRANB3*** ; *ENSCAFG00000005064*; ***R3HDM1***; *UBXN4*	‐	
19	39.5	39.5	0.03	2	3.23	2.91	n.a.	‐	
20	57.6	57.7	0.07	3	3.18	3.10	*ENSCAFG00000031730*; *ENSCAFG00000023991*; ***ARHGAP45***; *ATP5F1D*; *CIRBP*; *MIDN*; ***STK11***; ***SBNO2***; *POLR2E*	‐	
35	7.9	8.1	0.14	4	3.26	3.09	*BMP6*; ***TXNDC5***; ***BLOC1S5***; *ENSCAFG00000009583*; *ENSCAFG00000024482*	‐	
*Swedish population*								
4	44.3	n.a.	n.a.	1	3.09	n.a.	***ENSCAFG00000017171***	‐	
4	46.9	n.a.	n.a.	1	3.27	n.a.	*ENSCAFG00000028841*	‐	
4	50.0	50.2	0.15	4	3.09	2.90	***ATP10B***	‐	
4	52.5	n.a.	n.a.	1	3.47	n.a.	***CLINT1***	‐	
12	66.7	67.2	0.47	10	3.36	3.13	*GPR6*; ***WASF1***; ***CDC40***; ***METTL24***; ***DDO***; *SLC22A16*; *CDK19*	‐	
12	67.7	n.a.	n.a.	1	3.13	n.a.	***SLC16A10***	‐	
18	54.9	55.3	0.36	7	3.45	2.99	*LRRC10B*; *PPP1R32*; ***SYT7***; *PGA*; *DDB1*; ***VWCE***; ***ENSCAFG00000016314***; ***SLC15A3***; *CD5*; *VPS37C*; *CD6*	‐	
19	50.6	n.a.	n.a.	1	3.12	n.a.	***KIF5C***	‐	
24	42.4	42.5	0.05	3	3.33	3.05	*RBM38*; ***CTCFL***	‐	
36	30.1	30.6	0.05	6	3.11	2.82	*GULP1*; *COL3A1*; ***COL5A2***	‐	

*Note*: SNPs within 200 kb were summarized into selection signature regions.

a Number of top SNPs in region.

b Standardized absolute iHS of the peak SNP (in that region).

c Average standardized absolute iHS across the SNPs of a region.

d Genes located within and +/− 40 kb around selection signatures. Genes highlighted in bold include a SNP that belongs to the top 0.5% of the test statistic; all others are located within the region or +/− 40 kb around selection signatures.

e There were no phenotypic associations (behavior, coat color, or coat length) with FDR‐adjusted *P*‐value <.1 for markers located within the top 10 selection signatures within populations.

The genes located within or closest to the 10 most extreme values of iHS (positional candidate genes) identified within populations (Table 1) have been previously associated with behavior. Regarding those on Chr 19, variants in *TMEM163* (transmembrane protein 163) were associated with active behavior in an open‐field test involving cattle.^18^ However, *TMEM163* is also a functional candidate for physical features, for example, for eye width and depth^19^ and hair color^20^ in humans. *NCKAP5* (NCK associated protein 5) was also identified as candidate gene for temperament in cattle^21^ and has been associated with numerous neurological conditions in humans.^22^, ^23^, ^24^

The iHS peak on Chr 4 in the Swedish population points to the *CLINT1* (Clathrin Interactor 1) gene. This gene is reported to be among the top risk genes for the susceptibility to schizophrenia in humans^25^ and markers near *CLINT1* were suggestive peaks associated with barking tendency in a genome‐wide association study of behavior traits in Labrador retrievers.^26^

We conducted a gene list enrichment analysis with Enrichr^27^, ^28^ of the 256 and 338 genes that were located in and close to (within 40 kb of) the regions of the top 0.5% iHS in the UK and Swedish populations, respectively. No pathways were significantly enriched after accounting for multiple testing, however, Panther pathway analyses indicated nominally significant (*P* < .05) functional enrichment of several pathways for the UK population: “heterotrimeric G‐protein signaling ‐Gi alpha and Gs alpha mediated” (*P* = .01; genes: *GRK4*, *GRK7*, *RGS12*, *ADCY2*, *ADRA2C*, *DRD2*), “Alzheimer disease‐presenilin” (*P* = .02; *TRPC6*, *MMP7*, *MMP27*, *RBPJ*, *MMP20*), “heterotrimeric G‐protein signaling ‐Gq alpha and Go alpha mediated” (*P* = .02; *GRK4*, *GRK7*, *CACNA1A*, *RGS12*, *DRD2*), “ionotropic glutamate receptor” (*P* = .03; *CACNA1A*, *SLC17A8*, *GRIA4*), and “axon guidance mediated by semaphorins” (*P* = .03; *CRMP1*, *FYN*). All of these functions have been shown to be relevant for behavior among other functions, for example, heterotrimeric G proteins in mood disorders, as reviewed in Reference 29, ionotropic glutamate receptors for long‐term synaptic plasticity, as reviewed in References 30, 31 and semaphorins in neuronal structure, as reviewed in Reference 32. Nominally significant pathways for the Swedish population were “5‐Hydroxytryptamine degradation” (*P* = .003; *ALDH3A2*, *ALDH3A1*), “apoptosis signaling” (*P* = .01; *MAP2K3*, *CASP9*, *DAXX*, *BAK1*, *BIRC2*, *BIRC3*), and “Thyrotropin‐releasing hormone receptor signaling” (*P* = .03; *PLCE1*, *STX3*, *TRHR*). 5‐hydroxytryptamine (serotonin) is an important neurotransmitter and plays a key role in numerous behavioral disorders and characteristics, for example, depression^33^ and aggressiveness.^34^

### 2.3 Selection signatures between populations

Another approach to identify signatures of selection is the comparison of genetic variation (eg, allele frequencies or haplotype structure) between different populations. Accordingly, signatures of differential selection between the two GSD populations were analyzed employing three different tests: the fixation index (F_ST_), the cross‐population extended haplotype homozygosity (XP‐EHH), and differences between ROH (ΔROH_Prop_). F_ST_ was calculated to determine genetic differentiation between UK and Swedish GSD populations. Low genome‐wide genetic differentiation was detected for the single SNP‐based statistic (F_ST_ = 0.021 ± 0.029) and for the SNP window‐based statistic (F_ST_ = 0.021 ± 0.016), consistent with previous within‐dog‐breed estimates.^35^

We scanned the genome for regions of genetic differentiation within overlapping 1 Mb windows and found 17 distinctive peaks that comprise the top 1% window‐based F_ST_ values on Chr 1, 9, 20, 22, 24, 29, 30, and 32, with values ranging from 0.07 to 0.16 (Table S3). The highest F_ST_ value (0.16) was found for a region on Chr 24 (22.0‐24.5 Mb), which contains 46 genes. Among these genes are several with functions in physical characteristics and behavior, for example, *SPAG4* and *SUN5* involved in cytoskeletal anchoring, *NCOA6* involved in glucocorticoid and corticosteroid receptor signaling and *ASIP* and *RALY* associated with skin and fur pigmentation. Furthermore, seven members of the bactericidal/permeability‐increasing (BPI) fold‐containing (BPIF) superfamily of genes are located in this region (*BPIFB2*, *BPIFB6*, *BPIFB3*, *BPIFB4*, *BPIFA2*, *BPIFA3*, *BPIFA1*, and *BPIFB1*). It was shown that these genes play a role in the innate immune system and lipoprotein metabolism, but also in the brain's response to oxidative stress (aging), relevant for neuropsychiatric diseases.^36^ Interestingly, high F_ST_ for Labrador retriever populations differentiated based on their coat color and function (gundog and show dog) was also detected in the same region on Chr 24 (22.4‐22.8 Mb) in a previous study.^37^

While the F_ST_ statistic detects differences in allele frequencies between populations, the XP‐EHH test, an approach based on LD, is designed to detect regions that are fixed (or nearly fixed) in one population but remain segregating in the other population. Extreme high (positive) and low (negative) scores are indicators of a region under strong positive selection in the UK and Swedish population, respectively. The region including the SNP with the highest score (3.4) for the UK population was located on Chr 35 (11.0‐11.5 Mb) and contains three genes (*NEDD9*, *ADTRP*, and *TMEM170B)* (Table S3). The *NEDD9* (Neural Precursor Cell Expressed, Developmentally Down‐Regulated 9) gene has been shown to be associated to cognitive impairment in mice,^38^ *ADTRP* is important for vascular development and function in mouse and zebrafish^39^ and *TMEM170B* has been reported to be downregulated in TCGA human breast cancer data.^40^ The region with the highest absolute score (3.8) for the Swedish population was located on Chr 12 (3.6‐7.5 Mb). This region contains 59 genes; *RNF8* and *TBC1D22B* are closest to the SNP with the most extreme score. The ubiquitin gene *RNF8* (ring finger protein 8) plays a role in the immune system and has also been linked to autism; a recent study in *RNF8* knockout mice indicated a role of this gene in synapse formation and cerebellar‐dependent learning abilities.^41^ The function of *TBC1D22B* is largely unknown but it may encode a GTPase‐activating protein.

As a third approach to identifying differential selection between the populations, we identified the regions showing differences in extended homozygosity. To identify these selection signatures, we calculated the between‐population differences in runs of homozygosity (ΔROH_Prop_), which describes the difference in the proportion of dogs with an ROH of a specified length at a given SNP. The average ΔROH_Prop_ value across the genome was low (0.07 ± 0.06), indicating considerable overlap of ROH between the UK and Swedish populations. However, some regions with ROH were predominantly present in only one population (Table S3). The highest absolute ΔROH_Prop_ indicating selection signatures in the UK population were found on Chr 17 and 32: the ROH mapped to Chr 17 (8.3‐8.4 Mb) and Chr 32 (13.3‐13.4 Mb) were present in over 70% of the UK dogs but less than 40% of the Swedish dogs. The genes located in these regions are *GREB1*, *NTSR2*, and *LPIN1* on Chr 17, with no characterized genes in the Chr 32 region. The neurotensin gene *NTSR2* is involved in dopamine modulation and a SNP in this gene has been tested in a polygenic model of highly sensitive personality in humans.^42^ *LPIN1* plays a prominent role in lipid metabolism regulating adipocyte differentiation and coregulating other genes involved in lipid metabolism. The highest absolute ΔROH_Prop_ indicating selection signatures in the Swedish population was found on Chr 1: a ROH mapped to Chr 1 (24.7‐25.5 Mb) was present in 90% of the Swedish dogs but only in 42% of the UK dogs and contains the genes *LDLRAD4*, *MOXD1*, and *CTGF* (see below).

#### 2.3.1 Target regions for divergent selection signatures between populations

**FIGURE 4:**
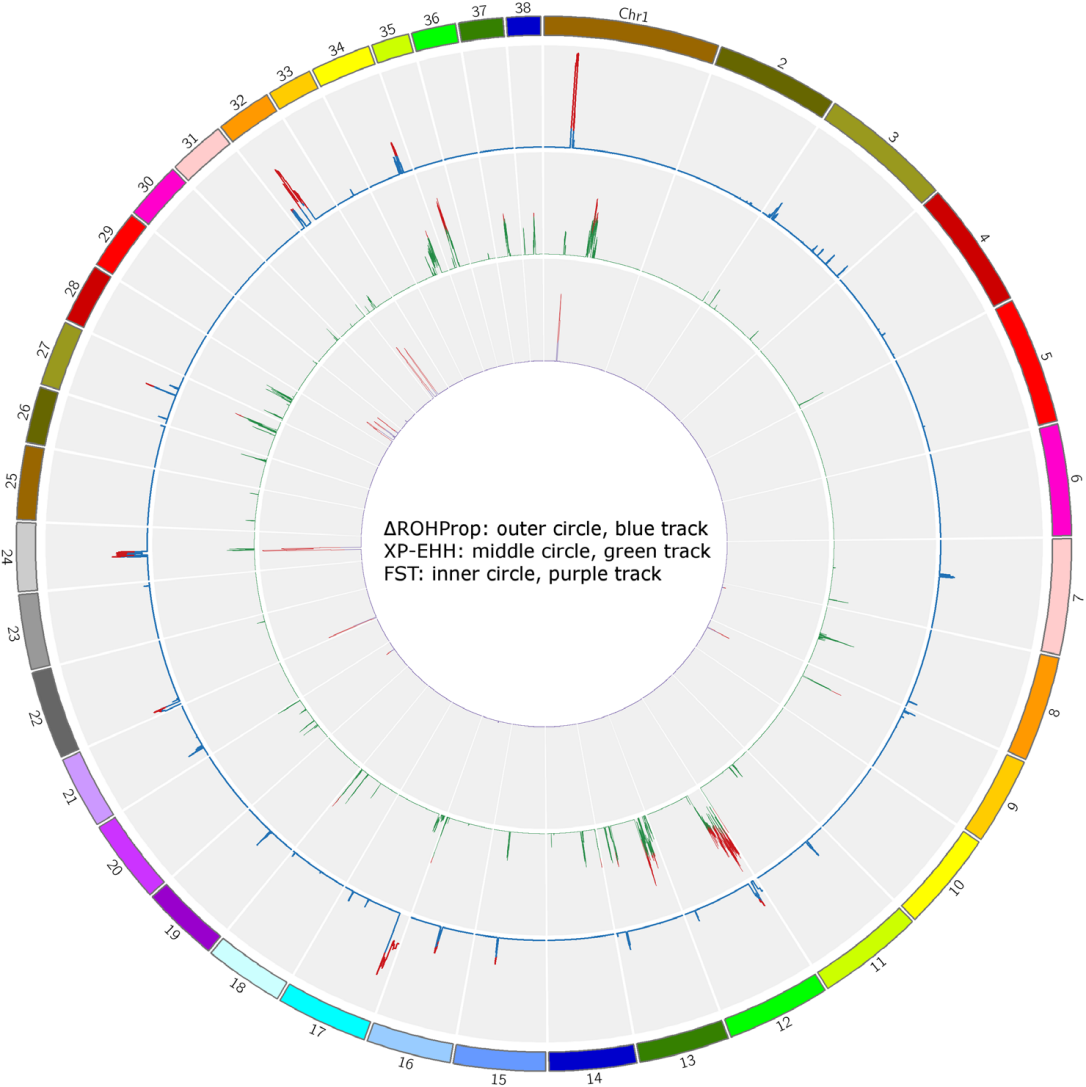
Circos plot for signatures of selection between GSD populations. The plot shows the three statistics used to identify regions under differential selection: differences between runs of homozygosity (ΔROH_Prop_, outer circle, and blue track), cross‐population extended haplotype homozygosity (XP‐EHH, middle circle, green track), and the fixation index (F_ST,_ inner circle, purple track). The plot indicates concordant evidence in regions on Chr 1, 24, and 32, where peaks can be seen based on all three methods (although not within the top 1% of SNPs for XP‐EHH, shown in red for the three methods)

In the detection of selection signatures, the application of multiple approaches is recommended to reduce the rate of false positive signals.^16^ To identify target regions under differential selection in the two GSD populations, we selected regions from the 99th percentile (top 1%) of each score distribution (SNP window‐based F_ST_, ΔROH_Prop_, and XP‐EHH) and searched for intersecting signals between two or three of the approaches. Using this criterion, we identified 433 SNPs (Table S3), with the greatest overlap between the SNP window‐based F_ST_ and ΔROH_Prop_ statistics (374 SNPs). No SNPs were detected by all three approaches. The 433 SNPs were located in 16 candidate selected regions on Chr 1, 9, 12, 22, 24, 32, and 34, which harbor 114 genes in total (Table 2; Figure 4). One Panther pathway was nominally significantly (*P* < .05) enriched by these 114 genes: “p53 pathway feedback loops” (*P* = .03; *CDKN1A*, *RBL1*). The SNPs identified as under divergent selection by these analyses were further tested for their association with different traits (coat color, coat length, and behavior) separately for each population to identify the putative trait under selection.

**TABLE 2:** Selection signatures that belonged to the top 1% of the distribution of at least two methods used to detect signatures of different selection between the GSD populations

Chr	Start	Stop	N_SNPs_ ^a^	Population	F_ST_ ^b^	ΔROH_Prop_ ^c^	XP‐EHH^d^	Gene(s)	Phenotypic association^e^
1	24 024 856	25 483 783	61	Sweden	0.12	0.46	NA	***ME2*; *MRO***; *MC2R*; *MC5R*; ***ENSCAFG00000000172***; *ENSCAFG00000029562*; *ENSCAFG00000029833*; *FAM210A*; ***LDLRAD4***; *ENSCAFG00000023012*; ***MOXD1***; *ENSCAFG00000031561*; ***CTGF***	Chasing*(UK)
9	16 472 361	16 493 753	4	UK	0.09	NA	2.81	*KCNJ16*; *KCNJ2*	‐
12	5 349 354	6 130 868	44	Sweden	NA	0.27	3.44	*BRPF3*; ***PNPLA1***; ***C12H6orf222***; ***ETV7***; ***PXT1***; *ENSCAFG00000001396*; ***KCTD20***; *STK38*; *SRSF3*; *CDKN1A*; ***ENSCAFG00000001418***; *ENSCAFG00000001419*; ***CPNE5***; ***PPIL1***; ***C12H6orf89***; *MTCH1*; ***PI16***; ***FGD2***	Stranger‐directed fear**(UK)
12	6 466 863	6 554 339	7	Sweden	NA	0.27	3.46	*FGD2*; ***CMTR1***; ***ENSCAFG00000030835***	Separation anxiety* (Sweden)
22	1 027 334	1 140 100	6	UK	0.08	0.26	NA	*RNASEH2B*	‐
22	1 683 950	2 496 568	46	UK	0.12	0.26	NA	***KCNRG*** ; ***TRIM13***; ***SPRYD7***; ***KPNA3***; *ENSCAFG00000031710*; ***EBPL***; *ENSCAFG00000010362*; ***RCBTB1***; ***PHF11***; ***SETDB2***; ***CAB39L***; ***CDADC1***; *ENSCAFG00000028525*; *MLNR*; *FNDC3A*	‐
24	22 002 778	22 463 326	24	UK	0.07	0.29	NA	*COMMD7*; ***DNMT3B***; ***MAPRE1***; ***EFCAB8***; ***SUN5***; ***BPIFB2***; ***BPIFB6***; ***BPIFB3***; ***BPIFB4***; ***ENSCAFG00000032553***; ***BPIFA2***; *ENSCAFG00000007369*; ***BPIFA3***; *BPIFA1*	Coat color**(UK)
24	22 908 179	23 816 844	37	UK	0.14	0.28	NA	*ENSCAFG00000029918*; *ENSCAFG00000007430*; *ENSCAFG00000007435*; *ENSCAFG00000029879*; *NECAB3*; *PXMP4*; ***ZNF341***; ***CHMP4B***; *EIF2S2*; ***RALY***; ***ASIP***; *ENSCAFG00000007508*; *AHCY*; ***ITCH***; *DYNLRB1*; ***PIGU***; *MAP1LC3A*; ***NCOA6***; *TP53INP2*	Coat color**(UK)
24	24 867 975	25 952 679	64	UK	0.13	0.28	NA	***CNBD2*** ; ***EPB41L1***; ***AAR2***; ***DLGAP4***; ***MYL9***; ***TGIF2***; ***SLA2***; *TGIF2‐C20orf24*; ***NDRG3***; ***DSN1***; ***SOGA1***; ***TLDC2***; ***SAMHD1***; ***RBL1***; ***MROH8***; ***RPN2***; *GHRH*; *MANBAL*; *SRC*	Coat color**(UK)
32	4 172 082	4 455 360	7	UK	0.09	0.27	NA	***ANTXR2*** ; ***PRDM8***	Coat length**(UK)
32	5 350 389	5 399 877	4	UK	0.13	0.26	NA	***PRKG2***	Coat length**(UK) and * (Sweden) Stranger‐directed aggression** (Sweden)
32	5 609 507	5 667 788	4	UK	0.12	0.26	NA	*ENSCAFG00000008928*; *RASGEF1B*	Coat length** (UK and Sweden)
32	13 000 437	14 125 551	44	UK	0.11	0.37	NA	***SNCA*** ; ***MMRN1***; ***CCSER1***	Coat color* (UK) Separation anxiety*(UK) Stranger‐directed aggression* (Sweden)
32	14 527 559	14 597 957	4	UK	0.11	0.38	NA	*ENSCAFG00000009954*	‐
32	14 952 127	15 194 499	4	UK	0.10	0.28	NA	*ENSCAFG00000009965*	‐
34	33 480 270		1	UK	NA	0.27	2.80		‐

*Note*: SNPs within 200 kb were summarized into selection signature regions. NA indicates that this selection signature was not present in the top 1% of the test distribution. Genes highlighted in bold include a SNP that belongs to the top 1% of the test distribution; all others are located within the region or +/− 40 kb around selection signatures.

a Number of top SNPs in region.

b Fixation index.

c Differences between runs of homozygosity.

d Cross‐population extended haplotype homozygosity.

e Significant phenotypic associations (behavior, coat color, coat length) for the UK and Swedish population within selection signature region. *P*‐values were adjusted using false discovery rate (FDR), with significant associations determined as adjusted *P*‐values <.05 (**) and suggestive associations as adjusted *P*‐values <.1 (*). The population for which the phenotypic association was identified is specified in parentheses.

A visual inspection of the Circos plot (Figure 4), which illustrates the results for the three approaches, indicates regions on Chr 1, 24, and 32 where peaks can be seen based on all three methods, although not belonging to the top 1% for XP‐EHH. Linear plots for these three regions illustrate the results from association analyses for traits with SNPs located in that region that have adjusted *P* < .1 (“Regional association”) and the selection signature test statistics (“Selection signatures”) (Figure S2). The specific population showing evidence of selection can be determined by the ΔROH_Prop_ or XP‐EHH score. Three regions showing evidence of selection in the Swedish population are located on Chr 1 (24.0‐24.1, 24.4‐25.1, and 25.3‐25.9 Mb; 17 genes), each harboring several interesting candidate genes. The *LDLRAD4 (*low‐density lipoprotein receptor class A domain containing 4) gene inhibits transforming growth factor‐β signaling^43^ and is a putative schizophrenia‐related gene.^44^ Another growth factor‐related gene in this region is *CTGF* (connective tissue growth factor). Other candidates for genes under selection in this region are the G‐protein‐associated melanocortin receptor genes *MC2R* and *MC5R*. *MC2R* (also known as the adrenocorticotropic hormone receptor gene, *ACTHR*) is a major modulator of glucocorticoid secretion regulation. *MC5R* has been associated with a range of phenotypes, including shedding and fur length in dogs,^45^ fatness in pigs, reviewed by Reference 46, and psychiatric disorders in humans.^47^ It was also differentially expressed in the brains of aggressive and tame foxes.^48^ These reported associations with different traits highlight one of the difficulties in identifying phenotypic targets of selection. In our analysis, we found no significant associations (FDR‐adjusted *P* < .05) between any of the selection signatures on Chr 1 with behavior traits, coat color, or coat length, but there was a suggestive association (FDR‐adjusted *P* < .1) with chasing behavior in the UK population (Table 2). Regarding fur shedding, GSDs as a breed are considered to be shedders, making it unlikely that there are large differences between the two populations for this trait.

Regions showing evidence of selection in the UK population are located on Chr 24 and 32. The Chr 24 candidate region under selection (22.9‐23.8 Mb; 18 genes) in the UK population comprises well‐known genes associated with black‐and‐tan and saddle‐tan coat color in dogs (*ASIP*, *RALY*).^49^, ^50^ We found highly significant associations in between coat color and SNPs in this region showing evidence of selection (Table 2, Figure S2). The saddle and tan/black and tan coat color was the dominant coat color in the UK GSDs while sable was predominant in the Swedish population (Table S1). The region on Chr 32 (5.4‐5.7 Mb; 3 genes) encompasses two behavior‐ and growth‐related candidate genes: *PRKG2* and *RASGEF1B*. *RASGEF1B* (RasGEF domain family member 1B) has been identified as a positional candidate gene for dog rivalry in a genome‐wide association study across multiple dog breeds.^51^ Several case studies have been carried out in humans on chromosomal diseases related to a microdeletion of loci homologous to the region on Chr 4 comprising the *PRKG2* and *RASGEF1B* genes.^52^, ^53^, ^54^ The loss of these genes leads to growth restriction, aggression, self‐injurious behaviors and mental retardation in affected individuals. The association analysis revealed a significant association between SNPs in this region and aggressive behavior toward strangers in the Swedish GSD population and *PRKG2* has previously been reported as a top candidate gene for anxiety in mice.^55^ However, the region on Chr 32 is in close proximity to the *BMP3* gene associated with skull morphology^56^ and the *FGF5* ^2^ gene associated with coat length in dogs. Regarding *BMP3*, differences in skull morphology have not previously been identified in GSDs nor have they been shown to carry a derived allele in this gene previously associated with brachycephaly,^56^ thus selection on skull morphology seems unlikely. However, we also found a highly significant association with coat length in both populations (Table 2, Figure S2), suggesting that this trait drives the selection signature on Chr 32 (via *FGF5*).

### 2.4 Which traits are under selection?

One of the main difficulties in interpreting genomic selection signatures is the identification of the actual trait(s) under selection. In dogs, the traits under selection are assumed to be primarily related to physical traits (eg, skull shape, coat color, body size) and/or behavior.^57^ While between‐breed studies have greatly contributed to the understanding of the genetic control of physical traits,^11^, ^58^ addressing behavior genetics by performing across‐breed selection signature analyses is likely to be challenging because breeds differ in multiple characteristics, including both behavior and these physical traits, many of which show Mendelian inheritance and thus tend to show very strong signals.

We employed several approaches to characterize the relationship between the detected selection signatures and phenotypic traits that were recorded for these populations. First, we repeated the ADMIXTURE analysis using only genotypes from SNPs identified as selection signatures (Figure S1) and fitted the ancestry assignment probabilities to the three individual clusters that were detected as factors in linear models for the phenotypes. We observed significant associations between UK (primarily associated with cluster 1) and Swedish (cluster 3) ancestries and some behavior traits (Stranger‐directed interest, Dog‐directed fear) (Table S4). Furthermore, highly significant associations were identified between the ancestries and other dog characteristics, including the function of the dog (working, pet or show dog), coat length, and coat color (Table S4). These results demonstrate a statistical association between these phenotypes and the dog's genotypes in the selection signature regions.

We then performed association analyses for behavior traits, coat length and coat color within each population only for markers within selection signature regions. We identified 87 SNPs with FDR‐adjusted *P* < .05 associated with coat length, coat color, human‐directed playfulness, stranger‐directed aggression, stranger directed fear, and dog‐directed fear (Table S5) in at least one of the populations. The striking significant associations for coat color (lowest FDR‐adjusted *P* = 3.37 × 10^−14^) and coat length (lowest FDR‐adjusted *P* = 1.13 × 10^−25^), comprising regions on Chr 24 and 32, respectively, have previously been identified for these traits^49^, ^59^, ^60^, ^61^ (Table 2).

As discussed above, previous studies on selection signatures in dogs have generally focused on inter‐breed or dog‐wolf comparisons and primarily detected selection signatures (and thus candidate genes) for physical features, for example, body size, coat characteristics, and skeletal morphology.^2^, ^11^, ^58^ Some studies, however, also identified signatures for neural crest development^1^ or brain function and nervous system development,^9^ which might be relevant for behavior especially in regard to domestication. We compiled a list of candidate genes reported in previous genomic analyses of phenotype associations and selection signatures in canids (dogs, wolves, foxes) focused on morphology and behavior and compared them with genes located in regions showing evidence of selection in our study (Table S6, note that the number of overlapping genes is not informative for identifying the trait under selection because the number of reported candidate genes differs substantially between studies). The biological functions of genes in common between the two lists are diverse and include a number of genes that have been associated with behavior. Major candidate genes for physical features in dogs, for example, *IGF1*, *SMAD2*, *FGF5*, and *BMP3*, as reviewed in Reference 7, were not detected within selection signatures in our study. However, *FGF5*, which has previously been associated with coat length, is located in close proximity to the selection signature on Chr 32 and we detected a highly significant association with coat length for this region (*BMP3*, associated with skull morphology, is also located near this region, but as discussed above, our data does not support a signature of selection associated with this trait). We also detected well‐described genes associated with coat color (Chr 24: *ASIP*, *RALY*). Together these results suggest that selection for morphological traits (coat length and coat color) has driven differences between the two populations in the genomic regions on Chr 24 and 32. In contrast, the region we detected on Chr 1 showed an association with Chasing in the UK population and comprises candidate genes with functions in behavior, but was not associated with morphological traits that we measured. Moreover, some of the selection signature regions showed associations with both morphological and behavior traits, for example, the region on Chr 32 was associated with both Stranger‐directed aggression and coat length in the Swedish population (Table 2). Furthermore, genes associated with physical appearance like *ASIP* have previously been associated with behavior traits, for example, social behavior in mice.^62^ Thus, it is possible that some of the selection signatures we detected are also associated with multiple traits.

### 2.5 Limitations of the study

By comparing UK and Swedish GSDs, we hypothesized that we would be able to detect selection signatures for behavior because behavior was the main selection target in the Swedish population. However, we found that the geographical origin of the dogs was confounded with other attributes, for example, coat color and length. We addressed the issue of which trait(s) were under selection by characterizing the relationship between selection signatures and associations with phenotypic attributes (behavior, coat length, coat color), recognizing that the sample size for the association analyses within populations was small and therefore these results should be interpreted with caution. In addition, measurements on other morphological traits (eg, body size and weight) were not available, but these might also be under selection and should be considered in future studies. We conclude that our study of GSD has identified selection signatures probably driven by selection for coat color and length (eg, at the *ASIP* and *FGF5* genes) as well as other signatures that may be related to differential selection for behavior between the Swedish and UK populations. Functional analyses are needed to test whether the identified candidate genes within regions showing evidence of selection do influence dog behavior characteristics.

## 3 MATERIAL AND METHODS

### 3.1 SNP genotyping and quality control

DNA was extracted from saliva samples collected with Performagene PG‐100 swabs (UK population) or blood samples (Swedish population). The genotyping was performed using the CanineHD Whole‐Genome Genotyping BeadChip^63^ featuring 172 115 SNPs. The data were filtered for sample call rate of >90%, SNP call rate > 98%, reproducibility (GTS) > 0.6 and low or confounded signal characterized by AB R mean (mean normalized intensity of the AB cluster) > 0.3 in GenomeStudio version 2.0. Minor allele frequency filtering of >0.01 was used to include rare but informative variants, leaving a final data set of 108 817 SNPs for analyses. Genotype information was available for 741 GSDs. Following further sample‐based quality control, closely related dogs were removed following the procedure described in Chen et al.^64^ Briefly, a pruned genotype data set to remove closely related dogs was created for SNPs with MAF > 0.05 using PLINK version 1.9^65^: based on the variance inflation factor, a function of the multiple correlation coefficient of a given SNP regressed on all other SNPs within a window (using default parameters: window size = 50 SNPs, overlapping SNPs for shifting windows = 5, the variance inflation factor threshold = 2). Then, GCTA version 1.24.7^66^ was used to compute the genetic relationship matrix and to remove one dog per pair with a genetic relationship higher than 0.2 (equivalent to second degree or closer relatives) leaving a final set of 182 UK and 68 Swedish GSDs for subsequent analyses.

### 3.2 Samples and phenotypes

The GSDs used in this analysis originated from the UK and Sweden. For the UK population, GSDs that were at least 2 years old and registered with the UK Kennel Club were recruited via email to participate in a study on behavior genetics.^14^, ^67^ GSDs from the UK population were bred by multiple breeders and primarily were pet dogs. All GSDs from the Swedish population were bred within the breeding program of the SAF starting in 2004 with the purpose of becoming working dogs. The strongest systematic selection pressure in the SAF breeding program is for behavior traits. Briefly, puppies were raised at the SAF, weaned at the age of 8 weeks and then fostered by members of the Swedish public.^68^ After a behavior test at the age of 15 to 18 months, some dogs started working with the SAF, Swedish Police or other authorities and companies, and/or were selected as breeding animals, whereas others were kept as pet dogs. For the Swedish population, owners, trainers, or handlers of GSDs bred within the breeding program of the SAF were invited via email or letter to participate in the study. Several phenotypes were analyzed. Data on GSD behavior was assessed using the Canine Behavior and Research Questionnaire (C‐BARQ).^69^ The C‐BARQ consists of questions related to training and obedience, aggression, fear and anxiety, separation‐related behavior, excitability, attachment, and attention seeking, and miscellaneous behaviors. To calculate the behavior traits, a PCA was applied to the data to condense the questions to a smaller number of 13 components, as described in Reference 14. The dogs' scores for the 13 components, adjusted for fixed effects (excluding cohort) as described in Reference 67, were considered as adjusted behavior traits in the subsequent analyses. Other dog characteristics (eg, sex, coat color, coat length, role) were assessed using a lifestyle survey.^14^ Summary statistics for behavior traits and other characteristics within the two GSD populations are given in Table S1.

### 3.3 Genomic structure of populations

To characterize the genomic structure of the GSD populations, a PCA and a cluster analysis were performed. PLINK version 1.9^65^ with default parameters was used to create a pruned SNP data set with reduced LD between SNPs, leaving a pruned data set of 9180 SNPs. This dataset was employed only to characterize the genomic structure of populations, via PCA and ADMIXTURE analyses. The PCA was performed in PLINK version 1.9^65^ and ancestry estimation was performed using ADMIXTURE version 1.3.0.^15^ The best number of clusters (K) was determined by comparing five‐fold cross‐validation (CV) errors.

Inbreeding, heterozygosity and nucleotide diversity were calculated within both GSD populations on the final data set of 108 817 SNPs. To determine inbreeding coefficients based on runs of homozygosity (F_ROH_), runs of homozygosity (ROH) were computed in PLINK version 1.9^65^ using the default settings of a ROH length of 1000 kb and a window size of 65 SNPs, as in Pfahler and Distl.^70^ The inbreeding was then estimated as the individual's total ROH length divided by the total genome length. ROH‐based methods have been shown to perform best in relation to the true inbreeding.^71^ Finally, nucleotide diversity (Nei's μ) was calculated per SNP using the –pi specifier in VCFtools.^72^

### 3.4 Identification of selection signatures

#### 3.4.1 Within populations

Signatures of selection within the two GSD populations were identified using the iHS statistic, which measures the EHH in the genome as an indicator of selective sweeps. The iHS statistic is based on the integrated EHH (iHH_*i*_), which is the integral of the observed decay of EHH away from a specified core allele *i* until the EHH reaches a specified cutoff. Phased genotypes of the final SNP data set generated by Beagle version 4.1^73^ (the phasing in Beagle was performed without specifying a reference population) were used to compute the SNP‐wise iHS statistic using hapbin,^74^ specifying that the iHH should be calculated up to the point at which EHH drops below 0.05 (−cutoff 0.05). As in Voight et al,^17^ the standardized iHS (iHS) for a SNP was calculated as

$$\mathrm{iHS}=\frac{\text{unstandardized}\;\mathrm{iHS}-\mu_{\text{unstandardized}\;\mathrm{iHS}}}{\sigma_{\text{unstandardized}\;\mathrm{iHS}}}$$

where the *unstandardized iHS* is ln(iHH_*i*_/iHH_*j*_) for alleles *i* and *j*, and *μ* and *σ* are the mean and the SD of the unstandardized iHS estimated from the empirical distribution of SNPs for which the derived allele frequency matches the frequency at the core SNP.

#### 3.4.2 Between populations

To detect divergent signatures of selection between populations, three different approaches were used: the fixation index (F_ST_), XP‐EHH, and differences between runs of homozygosity (ROH).

First, the F_ST_ analysis was performed using the script described in Talenti et al.^75^ The F_ST_ between UK and Swedish dogs was calculated for each SNP according to the formula reported by Karlsson et al,^76^ which is a comparison of the allele frequencies between populations:

$$F_{\mathrm{ST}}=\frac{f_{1}^{\mathrm{UK}}\left(f_{2}^{S}-f_{2}^{\mathrm{UK}}\right)+f_{1}^{S}\left(f_{2}^{\mathrm{UK}}-f_{2}^{S}\right)}{\left(f_{1}^{\mathrm{UK}}*f_{2}^{S}\right)+\left(f_{2}^{\mathrm{UK}}*f_{1}^{S}\right)}$$

where $f_{1}^{\mathrm{UK}}$ and $f_{2}^{\mathrm{UK}}$ are frequencies in the UK population for the two alleles and $f_{1}^{S}$ and $f_{2}^{S}$ are allele frequencies in the Swedish population. Next, the mean F_ST_ was calculated in 1 Mb sliding windows (window‐based F_ST_) with an overlap between windows of 500 kb, resulting in each SNP being located in exactly one or two windows. To derive a SNP‐based value (to select the top 1% for calculating the intersection with other methods as described below), we averaged the window‐based F_ST_ for the one or two windows in which the SNP was found.

Second, the XP‐EHH statistic^77^ was calculated to compare the EHH between populations, that is, whether alleles are homozygous in one population and polymorphic in the other population. The XP‐EHH statistic was calculated for the UK and Swedish populations using phased haplotypes generated by Beagle version 4.1^73^ in hapbin,^74^ as described above.

For the third approach, ROH were computed in PLINK version 1.9.^65^ We ran the analysis with the default settings of a ROH length of 1000 kb and a window size of 65 SNPs, as described above.^70^ For every SNP, a homozygosity score (ROH_Prop_) was calculated by dividing the number of dogs with a ROH at a specific SNP by the total number of dogs, such that ROH_Prop_ ranges from 0 to 1, as described in Bertolini et al.^78^ The absolute difference between ROH_Prop_ between populations (ΔROH_Prop_) was used as statistic to determine which ROH are highly represented in one population but underrepresented in the other population. Therefore, for every SNP, ΔROH_Prop_ values were calculated to identify ROH that are present in the majority of dogs in one population but not in the other.

#### 3.4.3 Gene identification and Gene ontology analysis

To detect putative genomic regions showing evidence of selection, the most extreme values from the test statistics were selected for both the within‐ and between‐population analyses to define selection signatures. For iHS, SNPs belonging to the top 0.5% of the distribution were selected. For F_ST_, XP‐EHH and ΔROH_Prop_, the top 1% of each test distribution was selected and the overlap between these top SNPs was determined to identify SNPs that had most extreme values for at least two of the three methods, to reduce the chance of false positive signals. We chose a less stringent threshold for top SNPs for between‐population statistics to allow for greater overlap since the three approaches differ in their methodologies and thus the ranking of top SNPs will vary. For a visual representation of target regions under selection between populations, the visualization tool Circos^79^ was used. For every SNP, the ΔROH_Prop_ and XP‐EHH scores were plotted. Since the F_ST_ was calculated as a window‐based average and Circos required a SNP‐based value, we averaged the window‐based F_ST_ for the one or two window in which the SNP was found, as described above.

The pairwise distances between the top SNPs were calculated and SNPs located within 200 kb were merged into a region. The distance of 200 kb was determined based on the LD in the genome. First, the squared correlation (*r* ^2^) between all pairs of SNPs within 10 Mb was calculated in PLINK version 1.9.^65^ The average *r* ^2^ was then calculated for bins of increasing distance between SNPs to identify the distance around SNPs at which average *r* ^2^ drops below 0.5. The longest bin for which average *r* ^2^ ≥ 0.5 was 200 kb.

To characterize functional relevance of regions showing evidence of selection, the top SNPs or regions (if multiple SNPs were found within 200 kb) were annotated for genes based on the CanFam3.1 genome assembly,^80^ using BEDtools 2.27 software.^81^ SNPs were annotated considering a flanking region of ± 40 kb, chosen based on the average between‐marker distance of the array (~20 kb), which was doubled to account for nonevenly spaced SNPs and SNPs lost through quality‐control filtering. The genes detected for these selection signatures were then submitted to Enrichr^27^, ^28^ to perform gene set enrichment analyses. Enrichr is an integrative web‐based application that compares submitted gene lists to various gene‐set libraries; the standard Fisher exact test option was used to calculate *P*‐values for this study.

### 3.5 Characterizing trait(s) under selection

We employed two approaches to gain insights into the trait(s) under selection, as detected as genomic selection signatures: (I) we modeled behavior traits and other dog characteristics as a function of the dog's ancestry based on selection signature regions and (II) we analyzed the association within each population between these traits and SNP markers in these regions. For both approaches, we compiled a genotype data set of SNPs within the regions showing evidence of selection; this included SNPs belonging to the top 0.5% of the iHS distribution in UK and Swedish populations and SNPs belonging to the top 1% of F_ST_, XP‐EHH, and ΔROH_Prop_ distributions that overlapped between at least two methods.

For (I), we repeated the ADMIXTURE analysis as described above, but only used genotypes of SNPs from putatively selected regions to estimate the ancestry. Then, a linear regression was performed, as described in Reference 82, to model the relationship between the traits and ancestry assignment probabilities.

For (II), we analyzed the association between the traits and SNP markers within the regions showing evidence of selection, separately for each population. Behavior traits were adjusted based on other fixed effects as defined in the previous study^67^ and treated as quantitative traits, while coat color (“saddle tan,” “sable,” “black,” “other”) and coat length (“long,” “short”) were treated as categorical traits and not corrected for environmental factors. The association analysis was performed using GEMMA,^83^ fitting the genomic relationship matrix (based on 108 817 genome‐wide SNPs) as a random effect to account for population stratification. To correct for multiple testing, *P*‐values were adjusted using the false discovery rate (FDR).

## CONFLICT OF INTEREST

The authors declare no conflict of interest.

## AUTHOR CONTRIBUTIONS

**Juliane Friedrich:** Conceptualization, Data curation, Formal analysis, Investigation, Methodology, Project administration, Writing‐original draft, Writing‐review, and editing. **Andrea Talenti:** Methodology, Software, Visualization, Writing‐review, and editing. **Per Arvelius:** Data curation, Project administration, Resources, Supervision, Writing‐review, and editing. **Erling Strandberg:** Data curation, Project administration, Resources, Supervision, Writing‐review, and editing. **Marie J. Haskell:** Conceptualization, Funding acquisition, Project administration, Supervision, Writing‐review, and editing. **Pamela Wiener:** Conceptualization, Funding acquisition, Project administration, Resources, Supervision, Writing‐review, and editing.

## DATA AVAILABILITY

Genotype and phenotype data for the UK dogs is available under CC‐BY license from the Dryad Digital Repository.^84^ The data for the Swedish dogs are restricted by the SAF for reasons of national security.

## Supporting information


**Table S1** Description of German Shepherd dog populations. Summary statistics for behavior traits and other dog attributes within the UK and the Swedish GSD populations.
**Table S4.** Significance of associations between population attributes and genetic ancestries. The proportion of ancestries estimated by ADMIXTURE (cluster 1, cluster 2, cluster 3) based on markers located within selection signature regions were fitted as fixed effects in separate linear models to test their association with different response variables (population attributes: behavior traits, role of the dog, coat color and coat length). The P‐values for the respective models are shown in the table.
**Table S6.** Overlaps between genes located in selection signature regions and candidate genes for morphological traits and behavior reported in other studies. A list of candidate genes in canids was compiled using the following references^1^, ^2^, ^9^, ^10^, ^11^, ^26^, ^37^, ^45^, ^50^, ^51^, ^58^, ^61^, ^67^, ^76^, ^85^, ^86^, ^87^, ^88^, ^89^ and was compared to genes located in regions detected as selection signatures in this study.
**Figure S1.** Ancestry proportions of GSDs based on genotypes of SNPs from putatively selected regions assuming three underlying ancestries (K = 3 clusters) as revealed by ADMIXTURE. Each cluster is represented by a color and the length of the specific colored segment indicates the dog's proportion of membership in that cluster. The labels indicate the origin of the dog (Sweden or UK) and the coat color (1 = saddle tan, 0 = sable, black or others).
**Figure S2.** Fine‐mapping of target regions under divergent selection between German Shepherd dog populations. Particularly compelling regions that showed evidence of divergent selection in all three selection signature test statistics (SNP window‐based F_ST_, ΔROH_Prop_, and XP‐EHH) are located on Chr 1, 24 and 32. The plots illustrate the FDR‐adjusted P‐values from association analyses for phenotypic traits (behavior, coat color, coat length) (above, “Regional association”) and the selection signature test statistics (below, “Selection signatures”) for all SNPs in these regions. The plots were created using a modified R code from that of Saxena et al. 2007. ^90^

**Table S2** List of SNPs belonging to the top 0.5% of the iHS statistic in the UK and Swedish populations.

**Table S3** Lists of SNPs belonging to the top 1% of the F_ST_, XP‐EHH and ΔROH_Prop_ statistics and the SNPs that belonged to the top 1% for at least two methods.

**Table S5** Markers located in selection signature regions and showing significant associations (FDR‐adjusted *P* < 0.1) with phenotypic traits (behavior, coat color, coat length).
