# Supplementary material for: Unravelling selection signatures in a single dog breed suggests recent selection for morphological and behavioral traits
Source: Adv Genet (Hoboken). 2020 Aug 10;1(1):e10024. doi: 10.1002/ggn2.10024 (PMC9744541; doi:10.1002/ggn2.10024)

Appendices

Table A1 Description of German Shepherd dog populations. Summary statistics for behaviour traits and other dog attributes within the UK and the Swedish GSD populations.

		**UK**	**Sweden**
**Number**		182	68
**Behaviour traits**†	Stranger-directed aggression	0.29±0.88	-0.04±0.9
	Dog-directed aggression	-0.13±0.97	0.21±1.14
	Stranger-directed fear	-0.02±1.11	-0.13±1.1
	Human-directed playfulness	-0.2±1.08	0.23±0.77
	Excitability	0.06±0.95	0.17±0.99
	Separation anxiety	0.03±0.89	0.02±0.96
	Lack of obedience	-0.09±0.95	-0.02±0.85
	Stranger-directed interest	0.24±0.96	-0.08±0.79
	Attachment/ Attention seeking	-0.12±1	0.06±0.94
	Chasing	0.24±0.93	-0.3±0.97
	Non-social fear	0.11±1	0.17±1.15
	Dog-directed fear	-0.01±0.92	0.11±0.97
	Touch-sensitivity	-0.02±0.87	-0.04 ±1
**Sex**‡	Male	97 (53%)	38 (56%)
	Female	85 (47%)	30 (44%)
**Role**‡	Pet dog	152 (84%)	37 (51%)
	Show dog	22 (12%)	1 (1%)
	Show and working dog	2 (1%)	0
	Working dog	6 (3%)	19 (28%)
**Coat length**	Short	106 (58%)	31 (46%)
	Long	70 (38%)	35 (51%)
**Coat colour**‡	Saddle tan/ Black tan	125 (69%)	13 (19%)
	Sable	41 (23%)	32 (47%)
	Black	12 (7%)	16 (24%)
	Other	4 (2%)	7 (10%)

†Mean and SD
‡Count and percentage

Table A4 Significance of associations between population attributes and genetic ancestries. The proportion of ancestries estimated by ADMIXTURE (cluster 1, cluster 2, cluster 3) based on markers located within selection signature regions were fitted as fixed effects in separate linear models to test their association with different response variables (population attributes: behaviour traits, role of the dog, coat colour and coat length). The P-values for the respective models are shown in the table.

		Cluster 1	Cluster 2	Cluster 3
Behaviour traits†	Stranger-directed aggression	0.214	0.497	0.111
	Dog-directed aggression	0.324	0.750	0.395
	Stranger-directed fear	0.527	0.527	0.282
	Human-directed playfulness	0.242	0.797	0.280
	Excitability	0.670	0.795	0.573
	Separation anxiety	0.210	0.641	0.291
	Lack of obedience	0.694	0.733	0.570
	Stranger-directed interest	0.056	0.891	0.045*
	Attachment/ Attention seeking	0.213	0.262	0.475
	Chasing	0.535	0.082	0.120
	Non-social fear	0.992	0.081	0.406
	Dog-directed fear	0.027*	0.158	0.118
	Touch-sensitivity	0.746	0.503	0.998
Show dog		0.037*	0.851	0.027*
Working dog		4.61e-06***	0.580	1e-05***
Coat length		4.96e-05***	0.0914	0.001**
Coat colour		<2e-16***	0.979	<2e-16***

† Behaviour traits were adjusted based on other fixed effects as defined in a previous study[1]. However, the population was not fitted as fixed effect for all behaviour traits and coat colour not for Chasing because the ancestry might be confounded with these attributes.
*P < 0.05
**P < 0.01
***P < 0.0001

Table A6 Overlaps between genes located in selection signature regions and candidate genes for morphological traits and behaviour reported in other studies. A list of candidate genes in canids was compiled using the following references[1, 2, 9, 10, 11, 26, 37, 45, 50, 51, 58, 61, 67, 76, 85-89] and was compared to genes located in regions detected as selection signatures in this study.

Putative trait	Candidate gene†	Study
Athletic success	*ARFGEF3, **ASIP**, CACHD1, CPQ, GRK4, **HTT**, RGS12*	Kim et al. 2018[12]
Barking	*CLINT1*	Ilska et al. 2017[10]
Behaviour	*ANKRD27, CAB39L, CEP126, EBPL, EPB41L1, FNDC3A, FYN, **GRAMD1C**, **HTT**, KPNA3, LDLRAD4, LRP1B, NUDCD1, PREX2, RASGEF1B, RCBTB1, RFX1, SETDB2, SHISA6, SLC17A8, SORCS2, SPATA5, STIM2, TBC1D14, TOP3A, USF3, ZRANB3*	MacLean et al. 2019[5], Zapata et al. 2016[19], Friedrich et al. 2019[1]
Brain development	*CACNA1A, RAI1*	Pendleton et al. 2018[14]
Coat colour	***ASIP**, RALY*	Vaysse et al. 2012[17], Dreger et al. 2013[7], Freedman et al. 2016[8], Boyko et al. 2010[4]
Domestication	*RAB3GAP1*	Axelsson et al. 2013[3]
Fur length, shedding	***MC5R***	Hayward et al. 2016[9], Schlamp et al. 2015[16]
Tameness	*CCSER1, DEK, EPAS1, FOXN2, **GRAMD1C**, GRK7, ITCH, KDM1B, NAA50, NCAM1, NCOA6, PIGU, RNF7, SIDT1, SPICE1, TP53INP2, ZDHHC23*	Kukekova et al. 2018[13]
Weight	*R3HDM1*	Plassais et al. 2019[15]

†Candidate genes for multiple traits are highlighted in bold

Figure A1 Ancestry proportions of GSDs based on genotypes of SNPs from putatively selected regions assuming three underlying ancestries (K = 3 clusters) as revealed by ADMIXTURE. Each cluster is represented by a colour and the length of the specific coloured segment indicates the dog's proportion of membership in that cluster. The labels indicate the origin of the dog (Sweden or UK) and the coat colour (1 = saddle tan, 0 = sable, black or others).

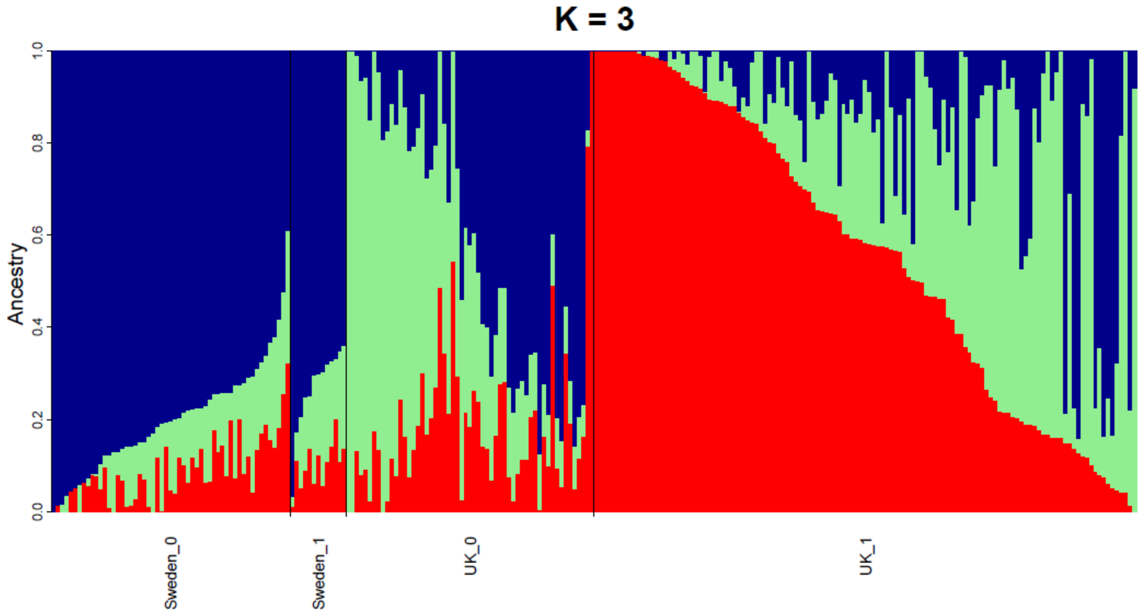

Figure A2 Fine-mapping of target regions under divergent selection between German Shepherd dog populations. Particularly compelling regions that showed evidence of divergent selection in all three selection signature test statistics (SNP window-based F_{ST}, ΔROH_{Prop}, and XP-EHH) are located on Chr 1, 24 and 32. The plots illustrate the FDR-adjusted P-values from association analyses for phenotypic traits (behaviour, coat colour, coat length) (above, "Regional association") and the selection signature test statistics (below, "Selection signatures") for all SNPs in these regions. The plots were created using a modified R code from that of Saxena et al. 2007[90].

Chromosome 1 (24.0 to 25.5Mb)

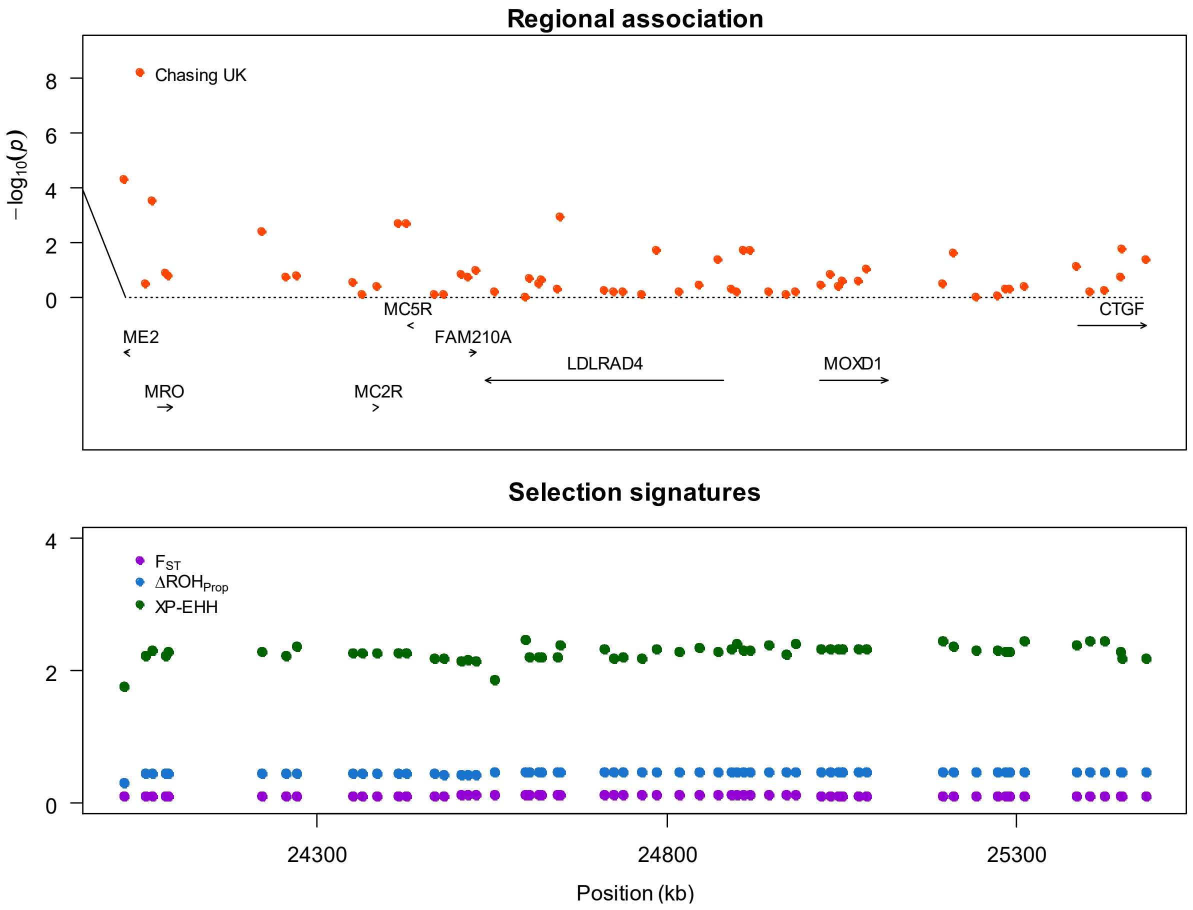

Chromosome 24 (22.0 to 26.0Mb)

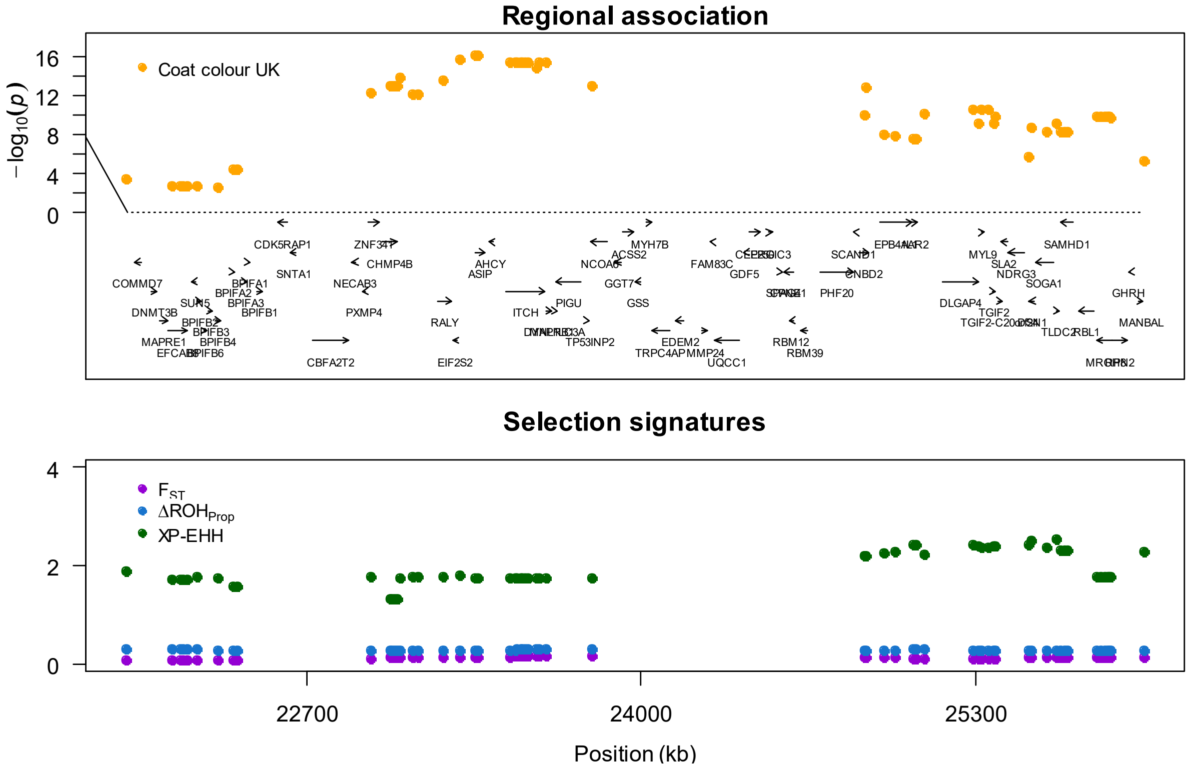

Chromosome 32 (53.5 to 56.7Mb)

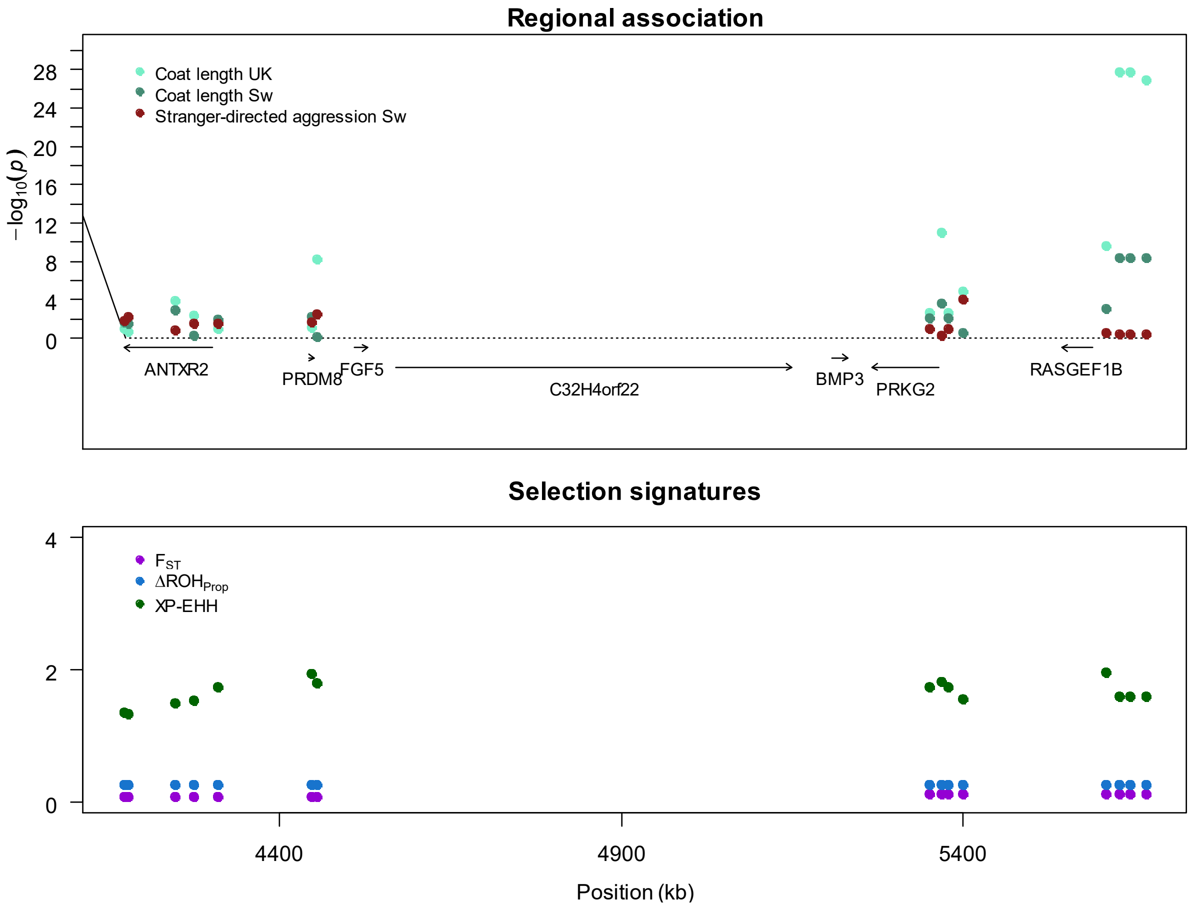

Supplement: Supplementary file 1 — Table S1 Description of German Shepherd dog populations. Summary statistics for behavior traits and other dog attributes within the UK and the Swedish GSD populations. Table S4. Significance of associations between population attributes and genetic ancestries. The proportion of ancestries estimated by ADMIXTURE (cluster 1, cluster 2, cluster 3) based on markers located within selection signature regions were fitted as fixed effects in separate linear models to test their association with different response variables (population attributes: behavior traits, role of the dog, coat color and coat length). The P‐values for the respective models are shown in the table. Table S6. Overlaps between genes located in selection signature regions and candidate genes for morphological traits and behavior reported in other studies. A list of candidate genes in canids was compiled using the following references 1 , 2 , 9 , 10 , 11 , 26 , 37 , 45 , 50 , 51 , 58 , 61 , 67 , 76 , 85 , 86 , 87 , 88 , 89 and was compared to genes located in regions detected as selection signatures in this study. Figure S1. Ancestry proportions of GSDs based on genotypes of SNPs from putatively selected regions assuming three underlying ancestries (K = 3 clusters) as revealed by ADMIXTURE. Each cluster is represented by a color and the length of the specific colored segment indicates the dog's proportion of membership in that cluster. The labels indicate the origin of the dog (Sweden or UK) and the coat color (1 = saddle tan, 0 = sable, black or others). Figure S2. Fine‐mapping of target regions under divergent selection between German Shepherd dog populations. Particularly compelling regions that showed evidence of divergent selection in all three selection signature test statistics (SNP window‐based FST, ΔROHProp, and XP‐EHH) are located on Chr 1, 24 and 32. The plots illustrate the FDR‐adjusted P‐values from association analyses for phenotypic traits (behavior, coat color, coat length) (above, “Region [file GGN2-1-e10024-s003.pdf]
